# Photonic resonator interferometric scattering microscopy

**DOI:** 10.1038/s41467-021-21999-3

**Published:** 2021-03-19

**Authors:** Nantao Li, Taylor D. Canady, Qinglan Huang, Xing Wang, Glenn A. Fried, Brian T. Cunningham

**Affiliations:** 1 Department of Electrical and Computer Engineering, University of Illinois at Urbana-Champaign, Urbana, IL USA; 2 Nick Holonyak Jr. Micro and Nanotechnology Laboratory, University of Illinois at Urbana-Champaign, Urbana, IL USA; 3 Carl R. Woese Institute for Genomic Biology, University of Illinois at Urbana-Champaign, Urbana, IL USA; 4 Department of Chemistry, University of Illinois at Urbana-Champaign, Urbana, IL USA; 5 Cancer Center at Illinois, Urbana, IL USA

**Keywords:** Nanostructures, Interference microscopy, Nanoparticles, Photonic crystals

## Abstract

Interferometric scattering microscopy is increasingly employed in biomedical research owing to its extraordinary capability of detecting nano-objects individually through their intrinsic elastic scattering. To significantly improve the signal-to-noise ratio without increasing illumination intensity, we developed photonic resonator interferometric scattering microscopy (PRISM) in which a dielectric photonic crystal (PC) resonator is utilized as the sample substrate. The scattered light is amplified by the PC through resonant near-field enhancement, which then interferes with the <1% transmitted light to create a large intensity contrast. Importantly, the scattered photons assume the wavevectors delineated by PC’s photonic band structure, resulting in the ability to utilize a non-immersion objective without significant loss at illumination density as low as 25 W cm^−2^. An analytical model of the scattering process is discussed, followed by demonstration of virus and protein detection. The results showcase the promise of nanophotonic surfaces in the development of resonance-enhanced interferometric microscopies.

## Abstract (web summary)

Here, the authors present photonic resonator interferometric scattering microscopy, which utilises a dielectric photonic crystal as the sample substrate. The resonant near-field enhancement leads to improved signal to noise ratio without increasing illumination intensity.

## Introduction

The detection and characterization of individual (bio)nano-objects directly in solution, across length scales that range from tens to hundreds of nanometers, with the high spatiotemporal resolution, and without the need for extrinsic labels or elaborate procedural steps, is the central utility of interferometric scattering microscopy. Demonstrations of biomolecular and nanomaterial mass determination^1,2^, sample heterogeneity analysis^3^, and dynamic molecular binding characterization^4^ have been achieved recently by interferometric imaging. In essence, the detected signal is generated by the interference between the object-scattered light and the background reference light, which scales linearly with the object size instead of the square dependence for pure scattering. As a result, interferometric signal contrast relies on the intensity balance between the molecule-induced scattered light and the reference laser beam^5^, apart from the scattering cross section (*σ*_sc_) of the molecule itself. To enhance the contrast, a common practice is to attenuate the overwhelming reference beam by a partial reflective mirror^6–8^, thin-film interference^9^, or polarization filtration^10^. However, high illumination intensity is needed for the molecule-scattered photons to overcome background noise. Alternatively, metallic surfaces have been employed to concentrate light by surface plasmon resonances to provide enhanced excitation for scatterers while drastically reducing the background^11^. The dissipative nature of the noble metal surface, however, leads to significant loss of scattered photons that are already scarce, especially in the case of scattering from small molecules. Similarly for noble metal nanoparticles, their contrast can be enhanced by exciting the localized surface plasmon as an alternative approach for enhanced excitation^12^.

In contrast to their plasmonic counterparts, nanostructured dielectric surfaces such as photonic crystals (PCs), can support a range of extraordinary optical properties without inherent material absorption loss^13^. As the optical analogs to electronic crystals, PCs consist of subwavelength periodic structures that resonate with photons, resulting in light confinement and photonic band structure. For instance, dielectric nanophotonic surfaces exhibiting certain topology symmetries can support high-quality-factor (*Q*) supercavity modes with a near-zero radiation channel to free space, also known as the quasi-bound state in the continuum (BIC)^14,15^. In addition, due to the strong correlation between the structure of the PC and its dispersion relation, PCs have been structurally tailored to realize various modulation transfer functions in the wavevector domain and employed for optical analog image processing, such as **k**-space filtering^16^, image differentiation^17–19^, and hyperspectral imaging^20^.

Here, we show that these combined characteristics of PCs yield a multifunctional platform for the implementation of interferometric scattering microscopy: the reference beam intensity can be significantly reduced by the photonic band edge, while the built-in optical resonances can be exploited to enhance light-matter interaction.

## Results

### Principle and simulation

**Fig. 1:**
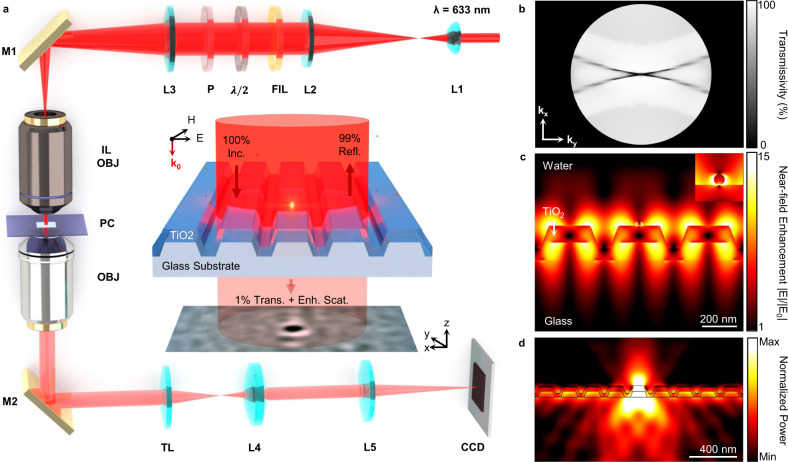
Principle of photonic resonator interferometric scattering microscopy (PRISM). **a** Sketch of the PRISM system, where an all-dielectric photonic crystal (PC) is used as the sample substrate and illuminated by a collimated 633 nm HeNe laser beam. Inset: normal-incident TM-polarized light excites the PC-guided resonance to enhance the particle-scattered light. The scattered photons then interfere with the 1% transmitted reference beam, resulting in an interferometric signal on the camera. L lens, FIL laser line filter, *λ*/2 quarter-wave plate, M mirror, OBJ objective, TL tube lens. The schematic images are not to scale. **b** Gray-scale encoded PC transmissivity (transfer function) at laser wavelength as a function of incident wavevector *H*(**k**_**x**_**, k**_**y**_) obtained from numerical simulation (NA = 0.70). **c** Normalized near-field electric field profile of a 20-nm diameter gold nanoparticle on the resonant PC substrate. **d** Normalized radiation power distribution of a vertically oriented electric dipole on the surface of a PC resonator.

Based on this hypothesis, we sought to develop photonic resonator interferometric scattering microscopy (PRISM) based upon an all-dielectric nanophotonic surface for the quantitative detection of individual nano-objects such as nanoparticles, viruses, and biomolecules. In place of an ordinary glass slide, a nanostructured PC surface (1 × 1 cm) is directly used as an optically resonant substrate in a transmission laser microscope (Fig. 1a). The PC consists of a TiO_2_-coated periodically corrugated polymer surface, fabricated on a coverslip by a low-cost replica molding process (Supplementary Fig. 1). When the incident monochromatic plane wave satisfies the phase-matching condition, the extended PC resonator will efficiently trap light through photonic crystal-guided resonance (PCGR), reshaping the optical near-field interaction and far-field propagation. The advantages of PCs in interferometric microscopy are threefold: (1) For laser excitation, PC functions as a notch filter centered in lieu of the partial reflective mirror. Near-unity back reflection and near-zero transmission can be obtained at resonance via a sharp Fano interference. For the transverse magnetic (TM) polarized plane wave at the wavelength *λ*_0_ = 63 nm, the corresponding modulation transfer function $H({\mathbf{k}}_{\mathbf{x}},\,{\mathbf{k}}_{\mathbf{y}})$ is obtained as the theoretical PC transmittance in the wavevector domain (Fig. 1b). Specifically, when the PC is illuminated by the collimated beam at normal incidence (Γ-point), <1% of the incident light is allowed to transmit and interferes at the image plane with the light scattered by nano-objects on the surface. As a result, the intensity of the reference beam is substantially reduced in the interferometric system. (2) By effectively trapping light in the resonant substrate, the PC provides nearly two-orders-of-magnitude enhanced excitation for the nanoscale scatterers via near-field coupling^21^ (Fig. 1c). This is achieved by the excitation of a pair of counterpropagating leaky modes in the corrugated TiO_2_ guiding layer, from which stationary wave patterns are formed, and a strong evanescent field at the water-TiO_2_ interface is induced. (3) The PC redistributes the light scattered from the nanoparticle angularly and thus improves the collection efficiency into the imaging objective lens. In addition to direct out-of-plane scattering, scattered light can be collected into in-plane guided modes, where it temporally recirculates in the PC resonator and eventually radiates into the lower substrate due to the nature of leaky modes (Supplementary Fig. 2). Through a point-dipole approximation for an individual NP, this scattering behavior can be more clearly demonstrated by the radiation power profile of a vertically oriented electric dipole on the PC surface (Fig. 1d). It is noteworthy that the radiated (scattered) light is predominantly (>78%) radiated into the substrate, whose refractive index (n_TiO2_ = 2.38) is much larger than that of the superstrate (*n*_water_ = 1.33). The PCGR-induced background suppression, enhanced excitation, and improved light extraction simultaneously enable the direct observation of nanoscale scatterers with a non-immersion (NA < 1) objective without significant loss of contrast signal at relatively low luminance. As a result, PRISM enables convenient noncontact-objective imaging and larger field of view.

### PC characterization

**Fig. 2:**
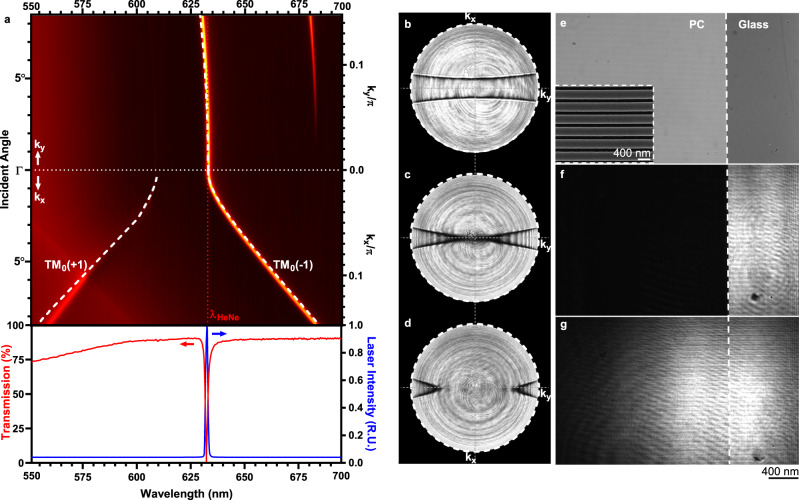
Characterization of photonic guided resonance mode. **a** Dispersion diagram measured experimentally from the transmission spectrum, overlaid with numerically obtained photonic band structure (dashed white curves). The laser wavelength *λ*_*L*_ = 633 nm is marked with a red dashed line, and the dotted horizontal line marks the normal incidence angle (Γ point). Lower panel: transmission spectrum (red) at normal incidence along with the normalized laser emission spectrum (blue). **b**–**d** Experimentally recorded isofrequency contour by Fourier plane imaging, respectively from PCs with TiO_2_ thickness of (**b**) 90 nm, (**c**) 97 nm, and (**d**) 105 nm. **e** Bright-field reflection image of the border between the PC nanostructure and the glass substrate. Inset: scanning electron microscopy (SEM) image of the corrugated PC surface. **f** PRISM image of the resonating PC sample used in (**c**). The majority of the normally incident light is reflected, leaving the PC region appearing black in transmission mode. **g** PRISM image of the detuned PC used in (**d**), where the PC region is almost transparent due to resonance wavelength mismatch.

To experimentally validate the physical principles underlying PRISM, the band structure of the fabricated PC is first obtained via a far-field transmission spectrum measurement (Fig. 2a). We also computed the band structure and derived the modulation transfer function $H({\mathbf{k}}_{\mathbf{x}},\,{\mathbf{k}}_{\mathbf{y}})$ using both finite-element method (FEM) and finite-difference method in time-domain (FDTD) to cross-validate the calculated results in the prevention of simulation artifacts (Methods and Supplementary Fig. 3). Quantitative agreement between the experimental results and the simulation modeling for the two branches of the PCGR mode (white dashed lines in Fig. 2a) are observed. Here, we focus on the long-wavelength resonance branch for its low transmissivity (0.764%) and high-quality factor (*Q* = 452.61). The PCGR resonance wavelength at normal incidence can be accurately tuned to match the HeNe laser wavelength (*λ*_0_ = 63 nm) by controlling the thickness of the deposited TiO_2_ layer (lower panel, Fig. 2a). To verify the modulation transfer function $H({\mathbf{k}}_{\mathbf{x}},\,{\mathbf{k}}_{\mathbf{y}})$, the angular diagram of the PC transmissivity at the laser wavelength is obtained through Fourier plane imaging. Figure 2c shows the obtained Fourier space image of a representative PC used in the PRISM system, where the isofrequency contour outlining the dispersive PCGR mode can be clearly observed, in good agreement with the predicted modulation transfer function (Fig. 1b). The measured modulation transfer function $H({\mathbf{k}}_{\mathbf{x}},\,{\mathbf{k}}_{\mathbf{y}})$ can be understood as a slice on the saddle-shaped photonic band (due to the anisotropic photonic lattice) in the wavevector plane $({\mathbf{k}}_{\mathbf{x}},\,{\mathbf{k}}_{\mathbf{y}})$ of constant frequency *ω*_0_ (red dotted line in Fig. 2a). For the detuned PCs, the photonic band is slightly shifted in the frequency axis and the corresponding isofrequency contours are therefore also offset from the saddle point (Fig. 2b, d).

One of the primary benefits of PCs in our microscopy system is that the background can be suppressed under normal incidence by virtue of the PCGR mode, without the need for illumination beyond the critical angle or additional modulation in the Fourier plane, therefore reducing the complexity of the imaging system. To demonstrate this capability, customized transmission illumination was added onto a conventional inverted microscope for the PC to be illuminated by a collimated TM-polarized beam from a 21 mW HeNe laser (Fig. 1a). For bright-field observation, the edge of the representative PC nanostructure was illuminated with 625 nm TM-polarized light from a light-emitting diode (LED), and inspected under reflection mode. Assisted by an oil-immersion objective (NA = 1.46), the horizontally aligned PC gratings (period Λ = 390 nm) are observable, and the brightness validates the high reflectivity of the PC in comparison with the adjacent glass substrate (Fig. 2e). When observed under transmission mode with laser illumination, the same PC region showed significantly lower background intensity than the adjacent glass substrate, as expected from the obtained transfer function in the wavevector domain. A slightly detuned PC, on the other hand, remains highly transmissive owing to the sharp Fano lineshape of the resonance mode.

### Calibration on contrast signal

**Fig. 3:**
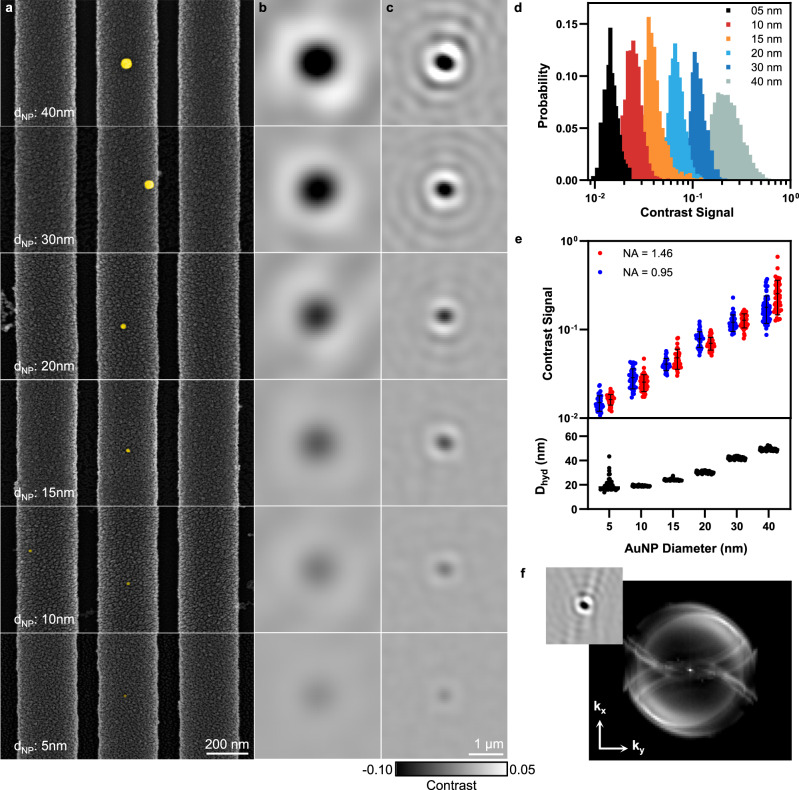
Imaging experiment with gold nanoparticles. **a** False-colored SEM images of a PC surface with AuNPs of various sizes. Correspondingly, differential interferometric images are recorded using (**b**) NA = 0.95 and (**c**) NA = 1.46 objectives for the same AuNPs under PRISM modality. **d** Contrast histogram of AuNP signal distribution obtained using a NA = 1.46 objective. (*n* > 500 for each size of AuNP.) The broadening of the contrast distribution for the 40 nm-AuNPs is likely caused by the formation of multiple-particle aggregates. **e** Scatterplot of the signal contrast as a function of AuNP size. Each dot represents the average of 50 instances of AuNP events. Lower panel: hydrodynamic diameters of the same AuNPs obtained through dynamic light scattering measurement. A clear separation of the hydrodynamic diameter can be except for AuNPs below 10 nm in diameter, where the instability of the laser becomes overwhelming comparing with the weak NP scattering signal. The DLS measurement for each AuNP size was repeated separately five times. **f** Fourier plane image of the AuNP (*d* = 40 nm) scattered light obtained by averaging the 2D Fourier-transformed images over 6000 frames. The outer circle is equivalent to NA = 0.95 in air. Inset: a typical real-space signal pattern of a 40-nm AuNP. FOV: 4.5 μm.

To explore the relationship between scattered signal image contrast and the size of the scattering object, we collected PRISM images of spherical gold nanoparticles (AuNPs) ranging in diameter from 5 to 40 nm. For the generality of results as applied to nanoparticles comprised of alternative materials, the laser wavelength is offset from the localized surface plasmon resonance (LSPR) wavelength of the AuNPs by at least 100 nm, preventing the synergistic coupling between the photonic resonator and the plasmonic resonator^22^. To verify the size of the AuNPs, both scanning electron microscopy (SEM) and dynamic light scattering (DLS) measurements were utilized as AuNP characterization. False-colored SEM images of AuNPs on the PC substrate are shown in Fig. 3a. For PRISM imaging, 20  μL of AuNPs solution (diluted to 1.0 × 10^10^ NPS/mL with molecular grade water) was dispensed on the PC substrate and sealed with a Piranha-cleaned coverslip, and individual AuNP binding/unbinding events were recorded at an acquisition rate of 600 frames-per-second (FPS). With the coherence-induced speckle background removed by rolling-window averaging^1^, representative images in Fig. 3b, c show the contrast signals from single AuNPs observed respectively with a 40× air objective (NA = 0.95) and a 100× oil immersion objective (NA = 1.46) (see “Methods”, Supplementary Figs. 4–5 and Supplementary Video 1 for details). A Laplacian-of-Gaussian (LoG) filter is applied to localize the center of every AuNP signal within each frame, followed by a single-particle tracking algorithm to obtain the lateral AuNP trajectories^23^ (Supplementary Note 1). The averaged contrast signal within each trajectory is considered as a single instance. From over 500 instances for each size of the AuNP recorded by the 100× objective, we constructed contrast histograms reflecting the distribution of interference signal dependent on the AuNP size/mass (Fig. 3d). In addition, the magnitude of the AuNP contrast signal recorded by the non-immersion 40× objective remains largely unchanged (Fig. 3e), indicating the scattering profile is confined to smaller angles, in contrast to the detection of a nanoscopic scatterer/emitter on a glass substrate where an immersion objective (NA ≥ 1.0) is required^24^. Interestingly, on top of a NA-dependent Airy disk pattern as commonly reported in the conventional interferometric microscopy, the real-space AuNP signal pattern also includes a semi-parabolic pattern (Fig. 3f inset), implying additional **k**-space information carried by the scattered photons. To extract the directionality as well as the angular distribution of the scattering profile of AuNPs on a resonant PC substrate, we performed Fourier transformation on over 6000 frames of the aforementioned interferometric image and obtained an averaged Fourier plane image (Fig. 3f). Despite the overlay of the shifted patterns caused by the laser-induced interference background, it is observed that a portion of the AuNP-scattered photons radiate into the far-field following the angular distribution described by the isofrequency contour $H({\mathbf{k}}_{\mathbf{x}},\,{\mathbf{k}}_{\mathbf{y}})$ in Fig. 2c. This result indicates that photons scattered from a NP can preferably couple into PCGR modes, consistent with the cavity-enhanced scattering reported elsewhere^25,26^. The isofrequency contours of these wavevectors are largely confined to the low-NA regime (NA ≤ 0.95), which explains the unaffected NP contrast signals by an air objective (Fig. 3e).

### Enhancement on scattering cross section

**Fig. 4:**
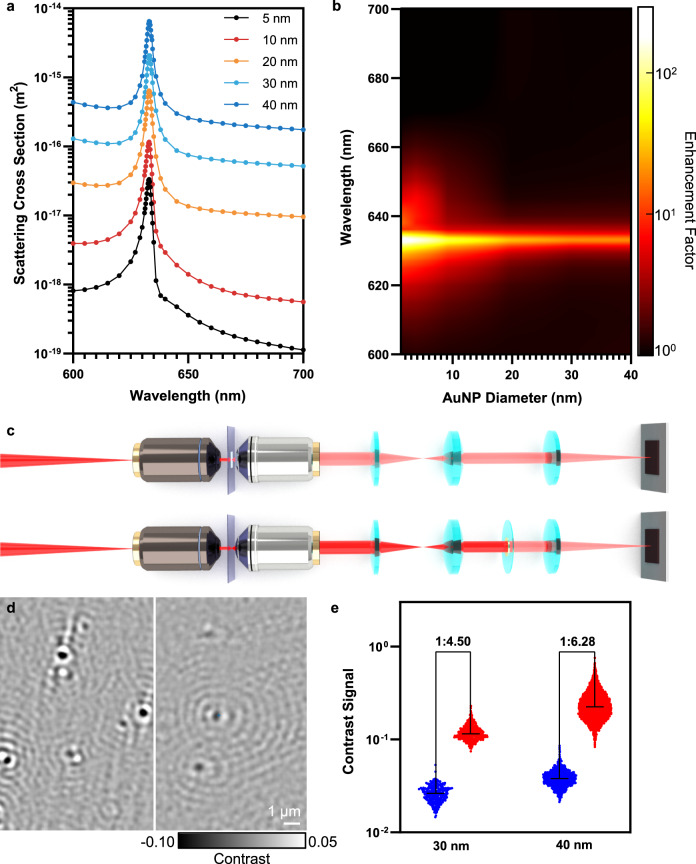
Modeling and validation on guided resonance-enhanced scattering. **a** FEM simulated scattering cross-section spectrum for AuNPs on a resonant PC surface. **b** Contour map of the PC enhancement factor on the AuNP scattering cross section as a function of AuNP diameter and excitation wavelength. **c** Schematic illustration of the PRISM system (upper) and interferometric scattering microscopy (iSCAT) with a gold disk placed in the Fourier plane to attenuate the reference beam (lower). **d** Representative differential interferometric images of AuNPs (*d* = 40 nm) obtained by PRISM (left) and iSCAT (right). **e** Comparison of signal contrast obtained respectively by PRISM (red) and iSCAT (blue). *n* > 400 for each size of AuNP.

The physical picture of the PC-nanoscatterer interaction can be delineated by temporal coupled-mode theory (TCMT)^22,27^ (Supplementary Note 2). The PC is treated as a resonator (resonant frequency *ω*_0_, radiative decay rate *γ*_*r*_), and allowed to couple with the non-resonant NP antenna (scattering damping rate *γ*_sc_). Here, absorption by the pristine PC resonator is neglected considering the low-loss property of dielectric material. The NP antenna is decoupled from free space radiation as its near-field interaction with the PC resonator is significantly stronger in comparison. Assuming a mirror-symmetry system, we obtain the resonator-mediated NP-scattered light as

1 $$\frac{{P_{sc}}}{{P_{in}}} = \frac{{2\gamma _r\gamma _{sc}}}{{\left( {\omega - \omega _0} \right)^2 + \left( {\gamma _r + \gamma _{sc}} \right)^2}}$$

where *P*_sc_ and *P*_in_ are respectively the scattered power and the incident power. From Eq. (1), it is indicated that the scattering signal follows a sharp Lorentzian lineshape centered near the PCGR resonant frequency. The scattering cross-section *σ*_sc_ for AuNPs obtained through FEM simulation exhibits good agreement with our analytical prediction, with a broadband background attributed to the onset of the AuNP plasmonic resonance mode (Fig. 4a). It can also be observed from Eq. (1) that the NP scattering efficiency is maximized when the radiative decay rate of the PC resonator matches the effective damping decay rate of NPs ($\gamma _r = \gamma _{sc}$). In comparison with a layer of solitary AuNPs, the PC-enabled scattering cross-section enhancement ratio at resonance can be obtained as^28^

2 $${{\Lambda }}({\upomega}_0) = \frac{{2\lambda _0\alpha }}{{\pi nd_e}}\frac{{\gamma _r}}{{\left( {\gamma _r + \gamma _{sc}} \right)^2}}$$

where *λ*_0_ is the resonant wavelength, *α* is the energy confinement of the PC mode in the NP layer, *n* is the refractive index of water and *d*_*e*_ is the effective length of the evanescent field. As the intrinsic scattering power of the NP scales with the sixth power of its radius, the scattering damping rate *γ*_sc_ approaches the radiative decay rate *γ*_*r*_ with decreasing size; therefore, the PC-enabled scattering cross-section enhancement ratio is also a function of NP size. This volume-dependent relationship is best captured in Fig. 4b, where the amplification of AuNP scattering cross section by the resonating PC substrate is obtained in comparison with that of a glass substrate. In general, AuNPs exhibit at least one-order-of magnitude enhancement in the scattering cross section, but for smaller NPs (*d*_AuNP_ = 5 nm) the cross section can be amplified by as much as 287 times.

The scatter enhancement by the PC resonator is experimentally validated by the AuNP contrast comparison between the PRISM system and a conventional interferometric system (iSCAT), where coverslips are used as the reference substrate and a partially transmissive (T ≈ 1%) gold disk is placed at the center of the Fourier plane for the attenuation on the reference beam (Fig. 4c). Under the same illumination intensity (25 W cm^−2^) and frame rate (600 FPS), interferometric images of water-suspended AuNPs were recorded and compared to demonstrate the ability of the resonant substrate to amplify the scattering signal (Fig. 4d). The signal contrast, measured at the centroid of the scattering pattern over 400 individual instances, was significantly improved when imaging with the on-resonant PC as the substrate by PRISM (Fig. 4e). Only AuNPs of 30 and 40 nm in diameter are compared here since the contrasts of smaller AuNPs by the partially transmissive disk are overwhelmed by the background noise. In hindsight, the contrast of the interferometric signal can be described as follows^5^

3 $$C = 2\frac{{E_{sc}}}{{E_r}}\cos \phi$$

where *E*_sc_ and *E*_*r*_ represent the electric field of the scattered light and the reference light, while *ϕ* is the phase difference between *E*_sc_ and *E*_*r*_. Therefore, the scattering signals from the two microscopy systems can be directly compared as long as the reference beam intensity remains constant. Our FEM calculation results predict that the PCCR offers a 28-fold amplification on *σ*_sc_ for AuNPs (*d* = 30 nm, 40 nm), or a 5.29-fold enhancement in terms of scattered electric field intensity (Fig. 4b), which is in good agreement with the experimentally obtained contrast amplification (Fig. 4e). In addition, it is noteworthy that the PC enhancement on the AuNP scattering is dependent on the relative NP location within one PC period, where the edge of the PC ridge provides more scattering enhancement due to the higher near-field intensity of the PCGR mode at the edge (Supplementary Fig. 7). This location sensitivity explains the broadened PRISM signal distribution for AuNPs in comparison with the DLS measurement results (Fig. 3e) and with the iSCAT measurement (Fig. 4e).

### Detection of proteins and virions

**Fig. 5:**
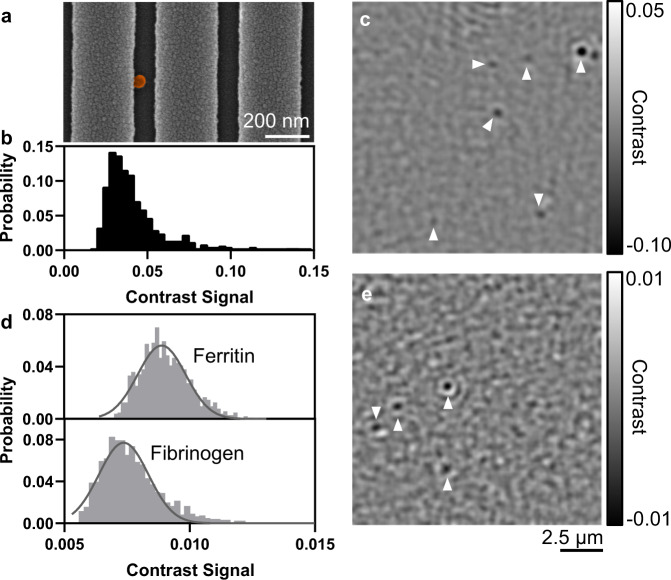
Application of PRISM to detect individual viruses and protein molecules. **a** False-colored SEM image of an inactivated SARS-CoV-2 virion on the PC substrate. **b** Contrast histogram obtained from over 500 individual virus signals. **c**, **e** Representative interferometric image with additional identifiers (white arrows) for coronaviruses and ferritin molecules within the field-of-view. **d** Distribution of contrast signal from individual ferritin (440 kDa, contrast: 0.88 ± 0.09%) and fibrinogen (340 kDa, contrast: 0.77 ± 0.09%). *n* > 1000 for each protein molecule.

As a demonstration of PRISM imaging of a biological nanoparticle, we evaluated the detection of individual SARS-CoV-2 viruses. The gamma (*γ*)-irradiated SARS-CoV-2 viruses were first imaged using SEM to characterize the virus morphology and physical dimension (*d* = 50.61 ± 7.97 nm, Fig. 5a). Diluted to 1  × 10^6^ pfu mL^−1^ with phosphate buffer saline (PBS) solution, the SARS-CoV-2 sample was directly introduced on the PC surface and observed under PRISM (Fig. 5b, Supplementary Video 2). The diffusion of virions was recorded within the field of view (14.6 ×  14.6 μm), and a high SNR contrast signals of ~ −4.55% was obtained from more than 500 virions (Fig. 5c). While the experiment records the presence of virions that transiently encounter the PC surface and their path along the PC surface during Brownian motion (see Supplementary Fig. 9 for the estimation of virion diffusion constant), we anticipate the use of selective capture molecules immobilized on the PC surface that can recognize outer surface features on the virus^29,30^, and bind the virus so that it will remain stationary.

Finally, to demonstrate the capability for PRISM to detect individual biological molecules, we exposed the PC surface to a solution containing large proteins. Ferritin (MW = 440 kDa) and fibrinogen (MW = 340 kDa) were prepared in buffer at concentrations of 100 nM and exposed to a bare PC substrate while recording PRISM image sequences at 600 FPS with 10-frame averaging. Representative images show individual protein molecules with contrast ranging from −0.5% to −1% (Fig. 5d). The histogram of signal intensity obtained from over 1000 transient surface scattering events reflects the separation of contrast distribution between the two protein molecules attributed to the mass difference (Fig. 5e). Since the background contrast is measured to be 0.20%, the detection limit of the current PRISM system is estimated to be 185 kDa.

## Discussion

We have demonstrated a type of interferometric scattering microscopy implemented by using a large-area PC resonator as the imaging substrate. The numerical simulation and theoretical modeling of the PC resonator and its interaction with nanoscale scatterers were complemented and confirmed by the experiment. It is demonstrated that by utilizing the all-dielectric PC substrate, three major benefits can be achieved simultaneously: low-transmission on the Γ-incident reference beam, resonance-guided angular scattering, and resonance-enhanced scattering cross section. These distinct advantages offered by PRISM allow for the real-time detection of individual biomolecules at relatively low illumination intensity, and the accurate quantification of the PC enhancement on the scattering signal for biomolecules requires further study. The PC nanostructures, produced by a low-cost replica molding process on ordinary glass coverslips, provide tunability in resonance wavelength, while the high-*Q* quasi-BIC resonance offers potential for further improvement on the scattering efficiency. In this study, the anisotropic property of the corrugated structure leads to the extension of the isofrequency contour into a higher NA regime, which can be further improved by a 2D lattice design.

Comparing with conventional interferometric scattering microscopy techniques, PRISM achieves the high-intensity excitation limited on the PC surface through light trapping. As a result, the contrast signal is broadened due to the non-uniform PCGR mode in the transverse direction, but can be addressed by employing a multilayer PC (1D PC), also known as the Bragg reflector, which consists of an alternating sequence of layers of low- and high-RI materials in the *z*-direction. The nanostructured resonator substrate introduces additional fabrication costs, but it also offers the interferometric scattering imaging modality to conventional laser transmission microscopes without modification on the optical path. Finally, although PRISM offers promises in utilizing a noncontact-objective for interferometric imaging without significant loss of contrast signal, by the Rayleigh criterion the maximum surface NP density will be compromised due to the lower NA. While the PC structures used in this report were fabricated in small batches in our research facility, we have also worked with a commercial vendor that uses holographic lithography to manufacture PCs of the same design on 8-inch diameter glass substrates at a cost of approximately $1 per sensor.

From a nanophotonic perspective, PRISM opens avenues toward single-molecule quantification, digital resolution measurement of biomolecular interaction dynamics, and diagnostic applications based upon detection of biological nanoparticles such as viruses and extracellular vesicles. It is expected that the enhancement on the particle scattering cross section can be further improved with the help of higher *Q*-factor resonators, or by the synergistic coupling between the scattering probes and the PC substrate^22^. In addition, exponentially decaying evanescent field offers approaches towards axial localization with higher accuracy in complement to the conventional PSF fitting methods.

## Methods

### Numerical simulation

Three-dimensional FEM and FDTD simulations were performed using commercially available packages (COMSOL Multiphysics and Lumerical FDTD). The fidelity of the fabricated PCs was validated by Focused Ion Beam SEM (Thermal Scientific Scios 2 DualBeam) where the cross-section profile is obtained, and the structure parameters are used in simulations (Supplementary Fig. 1). The unit cell in simulation consisted of one primitive PC period (390 nm) in the transverse direction imposed with Floquet periodic boundary conditions, while perfectly matched layer (PML) boundary conditions were imposed for the vertical direction. The refractive index of TiO_2_ was taken from Siefke^31^ while the refractive indexes of the glass substrate (*n*_glass_ = 1.510) and the UV curable epoxy (*n*_epo_ = 1.464) were referenced from the manufacturers. The band structure obtained from both FEM and FDTD shows good agreement with the experimental dispersion diagram (Fig. 2a). For FEM simulation of the resonance-enhanced scattering, the AuNP was modeled as a spherical structure with the refractive index provided by Johnson and Christy^32^. The mesh size at the NP is smaller than one tenth of the particle diameter. Using the wave optics module, the full field solution under Floquet periodic conditions was first calculated to simulate the field profile of a pristine extended PC resonator, which was then used as the background field for AuNP excitation and the previous Floquet periodic boundary conditions were replaced by PML boundary conditions to prevent the back-scattered field from the computational boundary. The detailed implementation is included in Supplementary Note 3.

### Device fabrication

The PCs were fabricated using a low-cost replica molding technique. A quartz molding template with the grating structure (390 nm period, 100 nm height) was fabricated using deep-UV lithography and reactive ion etching (Molecular Imprints). As the substrates for PCs, 20 × 60 mm coverslips (Electron Microscopy Science) were rinsed with acetone, isopropyl alcohol (IPA) and deionized water, followed by a piranha bath for more than 3 h. The piranha-cleaned coverslips were then rinsed with deionized water and dried under a continuous stream of nitrogen gas. After an oxygen plasma treatment at 500 mT and 200 W for 10 minutes, a layer of hexamethyldisilazane (HMDS, Shin-Etsu MicroSi) was spin-coated at 3000 rpm for 30 s, and baked at 100 °C for 1 min. Then, 5 μL of UV-curable epoxy was drop-cast on the quartz mold and covered by a pretreated coverslip, followed by a 40 s exposure under a 500 W UV lamp (Xenon RC-600). The cured replicas were gently lifted from the quartz mold and deposited with a layer of TiO_2_ (97-nm thickness) using a reactive RF sputtering system (Kurt Lesker PVD 75). The PCs were stored in a coverslip case filled with nitrogen gas. For fabrication of the gold disk attenuator, 25-μm-thick adhesive film (3 M 8211OCA) with a 2 mm hole at the center was attached to a piranha-cleaned N-BK7 optical window (Thorlabs WG11010). The masked N-BK7 window was deposited with 5 nm of Titanium followed by 100 nm of gold (Temescal Ebeam evaporator 4). With the adhesive stripped by acetone, the dot attenuator was measured to have a transmissivity of 0.65%.

### Spectroscopic measurement

For the measurement of PC transmission spectra under specific incident angles *θ*_inc_, the water-immersed sample was mounted on a fine-resolution rotation stage, with the PC gratings parallel to the rotational axis. Collimated TM-polarized white light from a tungsten halogen lamp (Ocean Optics LS-1) was incident onto the PC surface (illumination area ~5  mm^2^), with the incident angle adjusted by the motorized rotation stage. The zero-order transmitted light was collected by a fiber collimating lens and guided by an optical fiber into a spectrometer (Horiba iHR550).

### PRISM instrumentation

The PRISM system was custom-built on top of the body of a commercial inverted microscope (Zeiss Axio Observer 7). As illustrated in Fig. 1a, a collimated polarized laser beam from a 21-mW red HeNe laser (Thorlabs HNL210LB) was expanded by a relay lens group and filtered by a laser line filter (Thorlabs FL05632.8-1). A half-wave plate was used to control the polarization axis while a linear polarizer set the incident field to be TM-polarized. A doublet focused the expanded beam onto the back focal plane of an illumination objective (Olympus LMPLFLN50×), and the collimated beam from the objective directly impinged on the sample which was mounted on the sample holder. The partially transmitted laser beam, along with the object-scattered light, was collected by an objective (Zeiss Plan-Apochromat 40×/0.95, or Zeiss Plan-Apochromat 100×/1.46 Oil DIC), and focused on one of the side ports, which was relayed by a 1× lens group onto a charge-coupled device (CCD) camera (FLIR GS3-U3-23S6M), with a resolution of 57 nm/pixel and a field-of-view of 14.6 × 14.6 μm under the 100× objective. A compact power meter (Thorlabs PM16-120) was mounted on a side port for transmission power monitoring. Similarly, for the conventional interferometric scattering modality, a 1× relay lens group was mounted on the third microscope side port but with the aforementioned gold disk attenuator placed at the Fourier plane between the two lenses, and a CCD camera (FLIR GS3-U3-23S6M) captured the interferometric image. During imaging experiments, custom software was used for live streaming both the raw side port images and the differential interferometric images simultaneously to locate the correct focal plane. Once identified, the focal position was secured by an autofocus module where an infrared beam was constantly measuring the objective-to-surface distance.

### Fourier plane imaging

The TM-polarized collimated beam from a 0.8 mW red HeNe laser (Thorlabs HNLS008L) was condensed by an objective (Olympus RMS40X-PF) on the water-immersed PC substrate mounted on a rotational stage, while the transmitted light was collected by a lens group (NA ≈ 0.35) to project the Fourier plane on a CCD camera (FLIR GS3-U3-23S6M).

### Sample preparation and data acquisition

The surfactant stabilized AuNPs (Cytodiagnostics) for system performance characterization were used as received without further surface modifications and diluted to 1.0 × 10^10^ NPS/mL with molecular grade water. Ferritin and fibrinogen purchased directly from Sigma-Aldrich were diluted to 100 nM with PBS solution. *γ*-irradiated SARS-CoV-2 virions were requested from BEI Resources and stored at −80 °C. Freshly thawed SARS-CoV-2 stock solution was diluted to 5 × 10^6^ pfu mL^−1^ with PBS solution and immediately added onto the PC. For each interferometric scattering microscopy measurement, 20 μL of sample solution was directly added onto the PC surface and sealed with a piranha-cleaned coverslip. Clamped onto the sample stage, the PC was illuminated by the normal-incident laser beam and the transmitted power was measured to ensure the quality of resonance, followed by the autofocus procedure, and movies of 10 s duration were recorded.

### Image processing

All image analyses were performed in Matlab (MathWorks) with custom software based on a published single-particle tracking method^23^. In brief, the image processing procedure contains three major steps: (1) A rolling-window averaging method is applied to remove the background by the virtue of the dynamic measurement. With the window size defined as *N*, each frame of the raw images was averaged with the following *N*-1 frames, followed by a ratiometric process where every average image was divided pixelwise by the following average image. (2) Detection of individual scattering signals was obtained by the microscope point spread function, which was approximated by a 2D Gaussian function for practicality. For each frame of ratiometric image, a LoG filter is first applied to identify the local minima, followed by a 2D Gaussian function fitting. A *t*-test analysis is performed to select only the signals significantly higher than the background noise. Localization of signal centroids was determined with a LoG filter, which in combination with the Gaussian fitting method can achieve sub-pixel accuracy. (3) With the single-particle tracking algorithm, the AuNP trajectories were calculated by linking the detected signals in consecutive frames, under the assumption that one signal can at most link to one signal in another frame, and no merging or splitting is allowed. The detailed implementation is included in Supplementary Note 1.

## Supplementary information

Supplementary Information

Description of Additional Supplementary Files

Supplementary Video 1

Supplementary Video 2

## Data Availability

The data that support the findings of this study are available from the corresponding author upon reasonable request.
