# Supplementary material for: Photonic resonator interferometric scattering microscopy
Source: Nat Commun. 2021 Mar 19;12:1744. doi: 10.1038/s41467-021-21999-3 (PMC7979857; doi:10.1038/s41467-021-21999-3)
Supplement: Supplementary file 1 — Supplementary Information [file 41467_2021_21999_MOESM1_ESM.pdf]

Supplementary Information

Photonic Resonator Interferometric Scattering Microscopy

Nantao Li[†,1,2], Taylor D. Canady[†,2,3], Qinglan Huang[1,2], Xing Wang[2,3,4], Glenn A. Fried[3] and Brian T. Cunningham[*1,2,3,5]

[†] These authors contributed equally to this work.
[1] Department of Electrical and Computer Engineering, University of Illinois at Urbana-Champaign, Urbana, Illinois 61801, USA
[2] Nick Holonyak Jr. Micro and Nanotechnology Laboratory, University of Illinois at Urbana-Champaign, Urbana, Illinois 61801, USA
[3] Carl R. Woese Institute for Genomic Biology, University of Illinois at Urbana-Champaign, Urbana, Illinois 61801, USA
[4] Department of Chemistry, University of Illinois at Urbana-Champaign, Urbana, Illinois 61801, USA
[5] Cancer Center at Illinois, Urbana, Illinois, 61801, USA
[*] e-mail: bcunning@illinois.edu

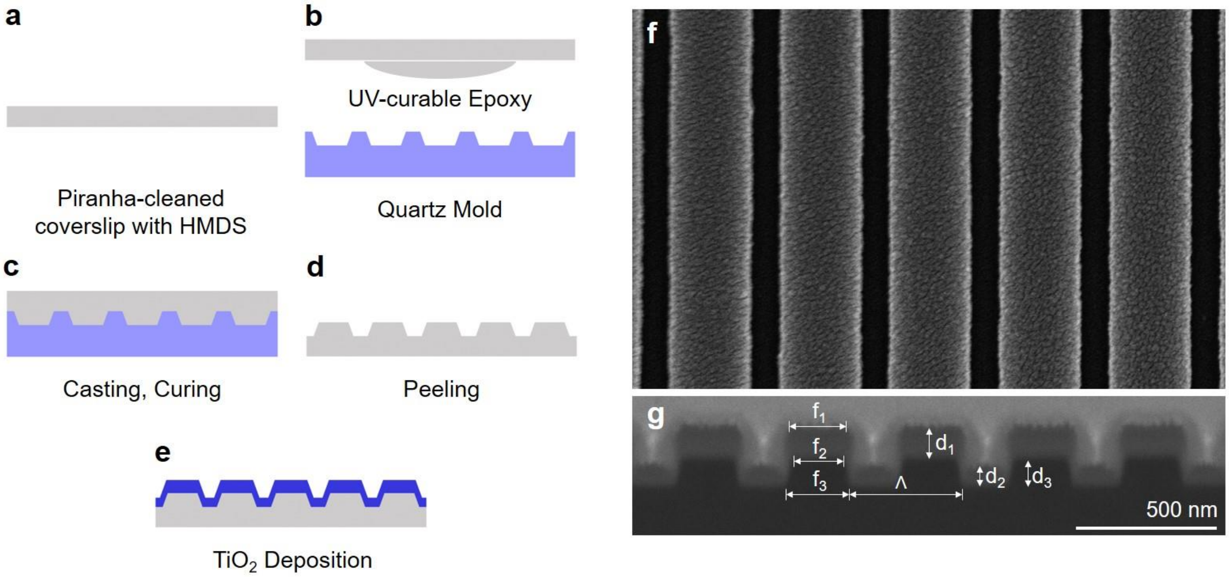

Supplementary Figure 1. | Fabrication and characterization of Photonic Crystal (PC) a-e, Schematics of the PC fabrication process, consisting of (**a**) piranha treatment followed by HMDS spin-coating for the coverslip substrate; (**b, c, d**) transference of the nanoscale grating on the quartz mold to the coverslip by a replica molding process where UV-curable epoxy is utilized to form the grating substrate on the coverslip once cured; (**e**) TiO_2 deposition on the cured polymer substrate to form the guiding layer in the PC design. (**f**) SEM and (**g**) FIB-SEM images show correspondingly the transverse and cross-sectional profile of the final PC structure, where the following parameters are extracted for numerical simulation: $\Lambda = 390$ nm, $f_1 = 0.60$, $f_2 = 0.55$, $f_3 = 0.74$, $d_1 = 97$ nm, $d_2 = 67$ nm, $d_3 = 100$ nm.

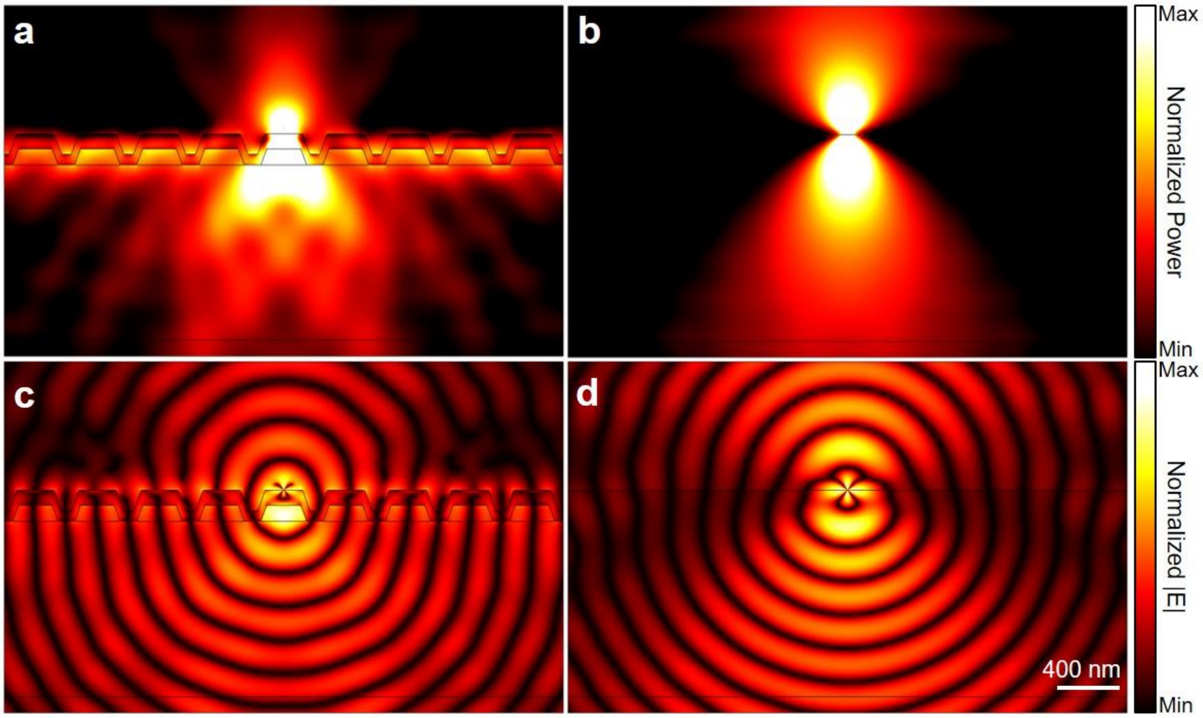

Supplementary Figure 2. | PC-assisted extraction on the electric dipole radiation. (**a**) Normalized radiation power distribution and (**c**) electric field magnitude of the radiated waves from a dipole place on top of the PC surface (with 0.5 nm separation). It can be observed that due to the match between the radiated photon wavelength (633 nm) and the PC periodicity, a portion of the dipole radiation power is coupled to the PC guiding layer, and subsequently leak into the substrate. The collection efficiency (78%) was obtained by first respectively integrating the radiation power within and below the PC structure, as well as the total radiation within the computation domain, the ratio of which is then obtained as the collection efficiency. In comparison, the conventional glass substrate (**b, d**) only offers a collection efficiency of 57%.

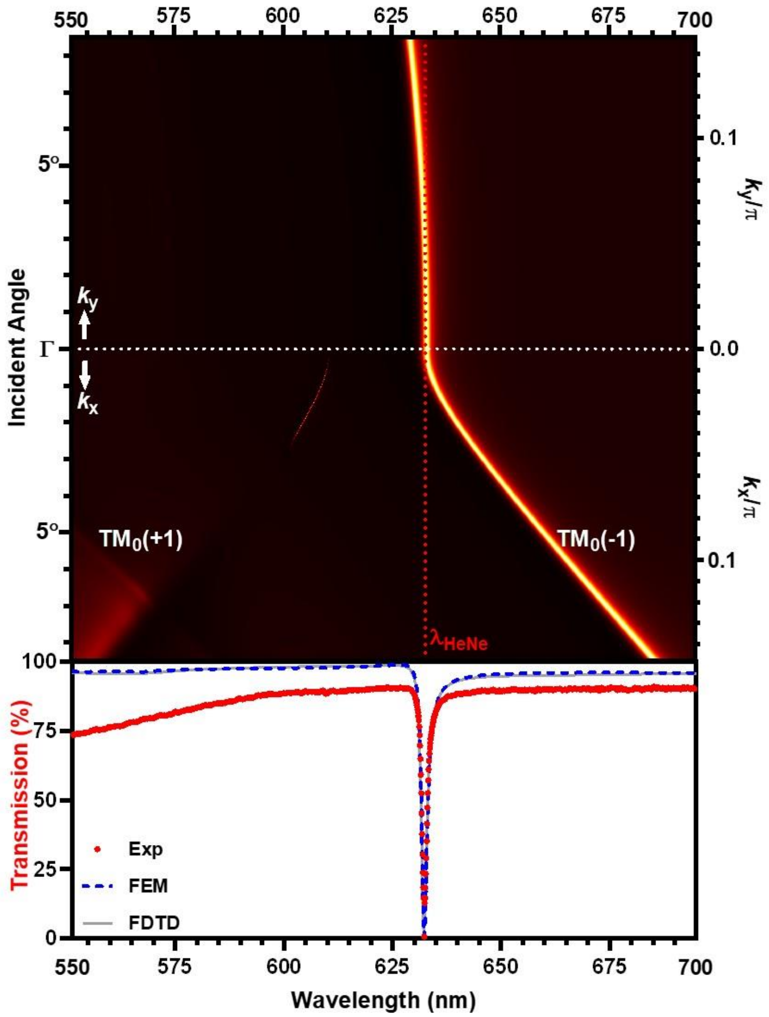

Supplementary Figure 3. | Numerical simulation on PC resonator. Based on the structural parameters obtained from SEM characterization, both finite element method (FEM) and finite-difference method in time-domain (FDTD) simulations were both used to cross-validate the PC performance. Upper panel: FEM calculated PC dispersion diagram, showing a good agreement with the experimentally obtained PC band structure shown in Fig. 2(**a**). Lower panel: comparison on normal incidence transmission spectra obtained both experimentally (red dotted line) and numerically: FEM (blue dashed line) and FDTD (gray line). The simulation results match closely to the measurement, except the slight background caused by interface reflection.

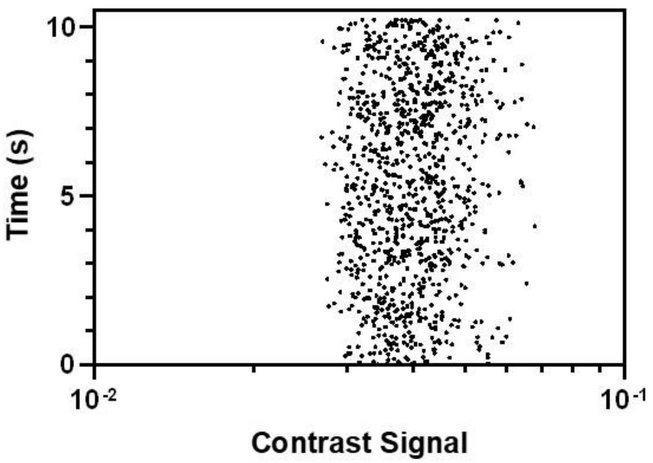

Supplementary Figure 4. | Scatter plot of signal AuNP events and their contrasts within a 10-s observation window. Each dot represents an individual AuNP (15 nm in diameter), and contrast intensity shows no dependency on time.

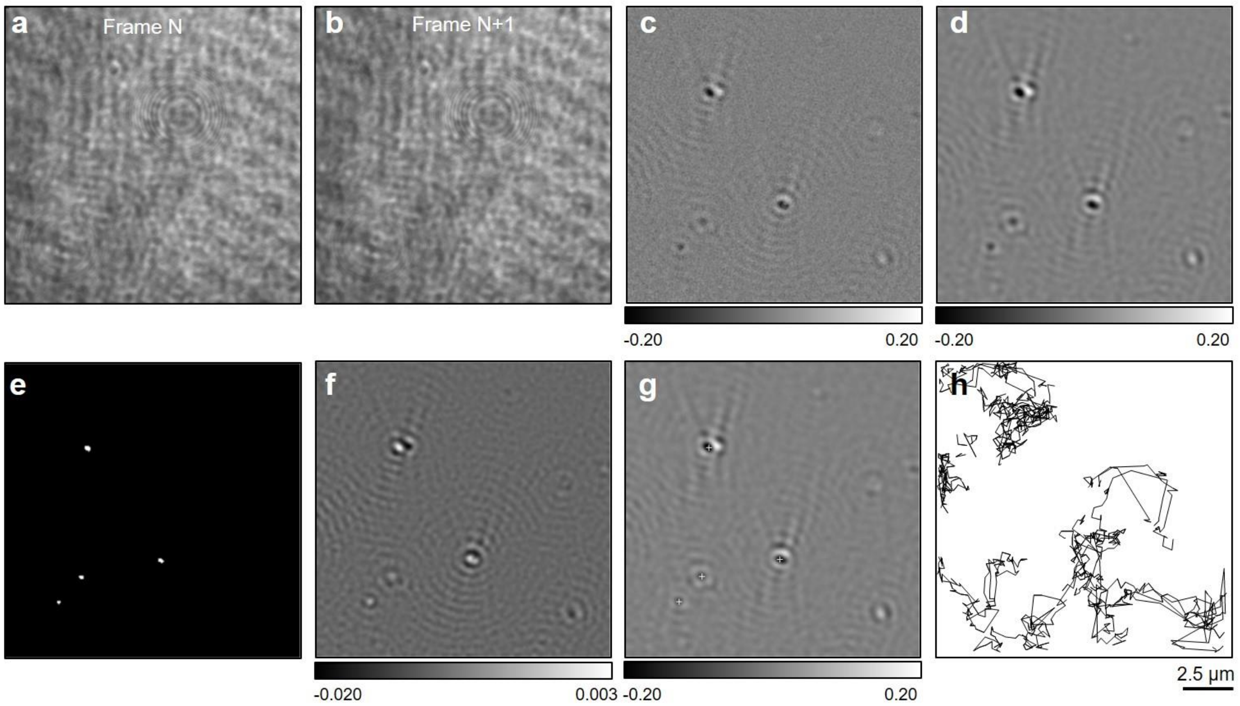

Supplementary Figure 5. | Image Processing for PRISM. (**a**, **b**) Two consecutive images with laser-induced speckle background before and after the arrival of AuNPs (40 nm in diameter). (**c**) Ratiometric image obtained by dividing (**a**) with (**b**). (**d**) Gaussian deconvoluted PRISM image in which background noise is significantly suppressed. (**e**) Calculated binary mask of significance indicating the pixels that follow the predefined PSF of the microscopy system. The centroids of interferometric signal are determined by locating the local minimum within each PSF patterns (marked with white cross in **g**), which can be obtained by overlaying the mask of significance with the local maxima of the Gaussian-of-Laplacian filtered image (**f**). (**h**) AuNP trajectories over consecutive frames are calculated by a Kalman filter. AuNP trajectories spanning over 500 frames (at 600 PFS) are shown in the figure.

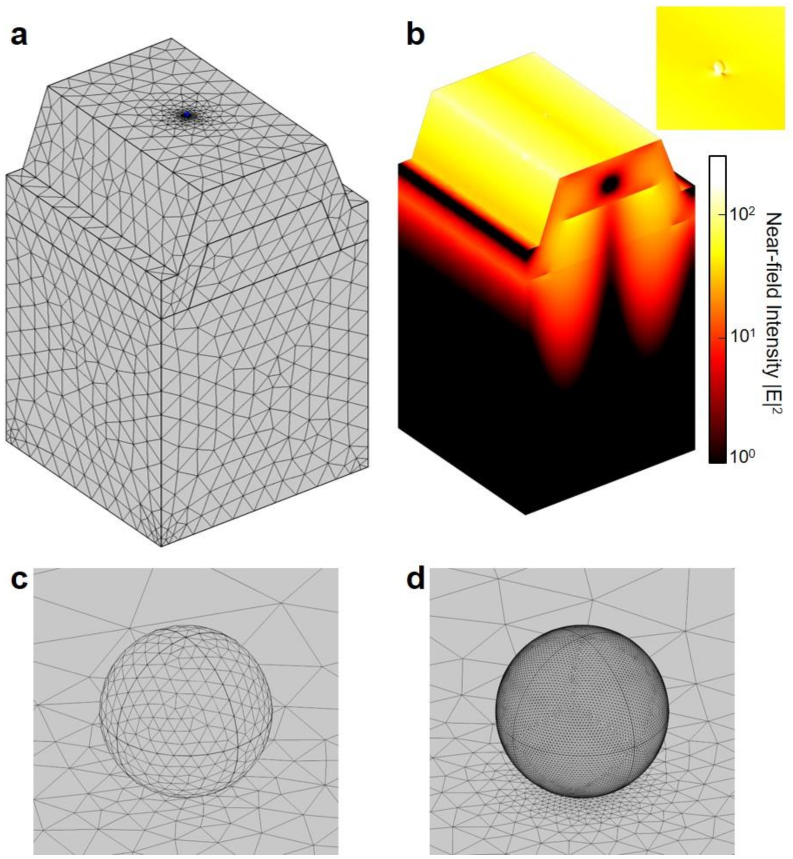

Supplementary Figure 6. | Mesh setting for FEM simulation and discretization error analysis. (**a**) A non-uniform mesh is applied for FEM simulation, where denser mesh is used to map the AuNP structure (mesh size less than one tenth of the AuNP diameter). For a AuNP of 5 nm in diameter, high density mesh (maximum element size is 0.5 nm) is in use to delineate the quick changes in electric field near the particle surface, while relatively coarse mesh is applied to the water layer and glass substrate. (**c**) The zoom-in image shows the mesh setting on the AuNP surface. Discretization error analysis is performed by further reducing the element size on the AuNP down to 0.1 nm (**d**), and the obtained near field intensity profile (**b**) shows less than 2% difference.

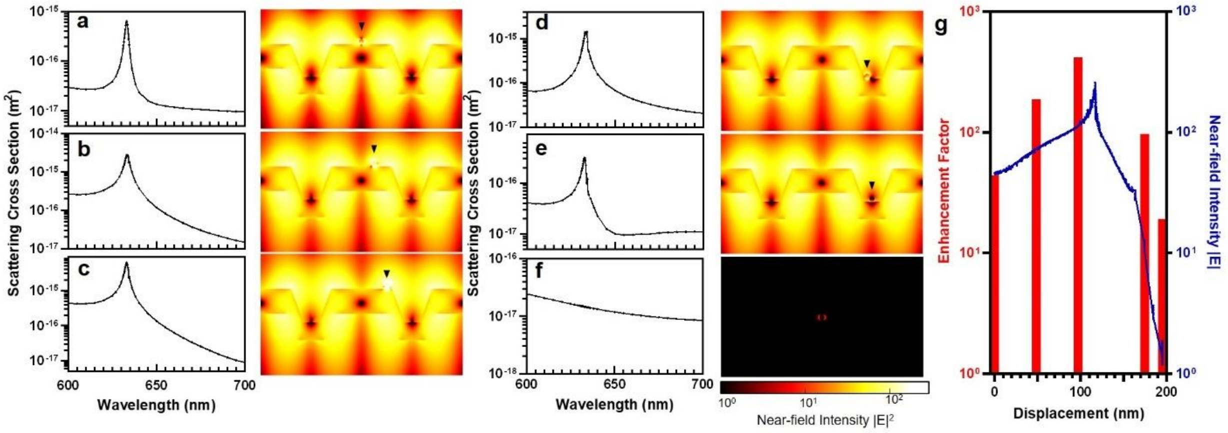

Supplementary Figure 7. | The effect of relative location of the AuNP on a PC substrate.

The scattering cross section spectrum and the normalized near-field profile for a 20-nm-diameter AuNP located respectively (**a**) at the ridge center, (**b**) at $\Lambda/8$ displacement from ridge center, (**c**) at $\Lambda/4$ displacement from ridge center, (**d**) at the groove corner, and (**e**) at the groove center. In comparison with a glass substrate (**f**), the PC provides at least one-order-of-magnitude enhancement on AuNP scattering even at the anti-node of the PCGR mode. (**g**) The location sensitivity of PC enhancement on scattering is closely related to the PCGR near-field profile, where maximum enhancement is achieved at the edge of the PC ridge.

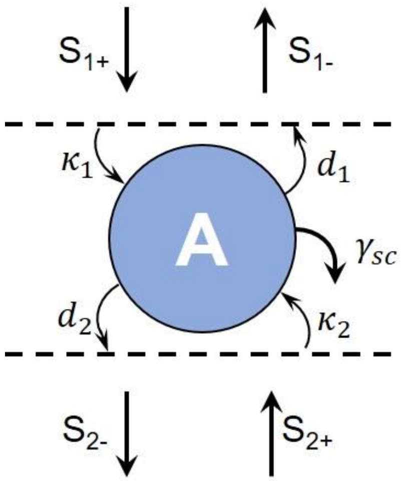

Supplementary Figure 8. | Schematic of TCMT model for PC assisted NP scattering. The PC cavity mode is characterized with mode amplitude *A* (resonant frequency ω_0, with radiative decay rate γ_r), and exchanges energy with incoming ($s_{(1,2)+}$) and outgoing ($s_{(1,2)-}$) waves ($s_{2+} = 0$) with coupling constant $\kappa_{(1,2)}$, $d_{(1,2)}$ respectively. The NP induced Rayleigh scattering is modelled with a damping rate γ_{sc} on the PC cavity mode.

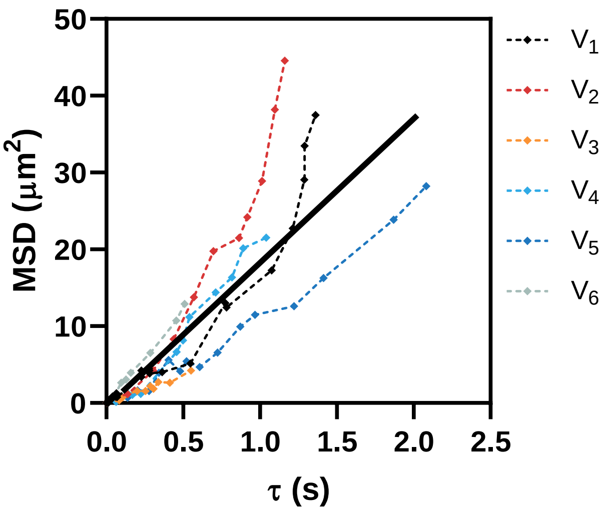

Supplementary Figure 9. | Mean-squared displacement (MSD) versus *τ* for SARS-CoV-2. Six separate SARS-CoV-2 virions (V_1-V_6) were respectively traced and their MSDs calculated. The averaged diffusion constant is found to be 10.24 $\mu m^2/s$, with a standard deviation of 4.51 $\mu m^2/s$. The estimation on the SARS-CoV-2 diffusion constant falls within the expected value for the Brownian motion of virus in water at 20°C.

Supplementary Note 1: Image Processing and Data Analysis

The image processing algorithm used in photonic interferometric scattering microscopy (PRISM) was conducted in Matlab (MathWorks) adapted from previously reported methods[1,2]. All the captured frames obtained from the camera were saved as individual raw images, for a typical observation window of 10 s, more than 6000 raw images were recorded for later analysis.

The image processing procedure contains three major steps:

(1) A rolling-window averaging method was applied to remove the background by the virtue of the dynamic measurement. With the window size defined as N, each frame of the raw images was averaged with the following N-1 frames, followed by a ratiometric process where every average image was divided pixelwise by the following average image. For the gold nanoparticle (AuNP) characterization as well as SARS-CoV-2 virion detection, we simply set the rolling window size as 1. Therefore, the ratiometric image is simply the direct division of two consecutive frames, as shown in Fig. S5 (**a-c**). However, the window size for protein detection is determined as 10 in order to suppress the background noise.

(2) The diffraction-limited PRISM signals are most accurately detected by point spread function (PSF) deconvolution, as commonly applied in super-resolution microscopy techniques such as PALM and STORM[3]. For simplicity, the airy-disk PSF function is usually approximated as a 2D Gaussian function for in-focus scatterers/emitters:

$$\mathrm{g}(\mathbf{x};\ \boldsymbol{\mu}) = \exp(-((x_1 - \mu_1)^2 + (x_2 - \mu_2)^2)/2\sigma^2) \tag{1}$$

where $\boldsymbol{x} = [x_1, x_2]$ is the spatial coordinate within the image, $\boldsymbol{\mu} = [\mu_1, \mu_2]$ is the coordinate of the location of the point source, and the standard deviation σ is a

predetermined parameter by the objective in use. Therefore, the ratiometric images were modelled as the PSF-convoluted images with additional Gaussian noise:

$$\mathrm{h}(\mathbf{x};\ \boldsymbol{\mu}, A, c) = Ag[\boldsymbol{x};\ \boldsymbol{\mu}] + c + n[\boldsymbol{x}] \tag{2}$$

where *A* and *c* are respectively the amplitude and the background intensity of the interferometric scattering signal, while $n[\boldsymbol{x}]$ is the background Gaussian white noise which follows *N*(0, σ_n^2). Consequently, the estimation on *A* and *c* on each candidate signal location $\boldsymbol{k} = [k_1, k_2]$ within each specific ratiometric image $f[\boldsymbol{x}]$ obtained in step (1) can be implemented by

$$\min v = \min \sum_{\mathbf{x} \in \mathrm{S}} (\mathrm{h}(\mathbf{x};\ \boldsymbol{\mu}, A, c) - f(\boldsymbol{k} - \boldsymbol{x}))^2 \tag{3}$$

where *S* is the 4σ area centered at the candidate location $\boldsymbol{k}$. Here, the candidate locations of scattering signals are obtained from the local minimum of the Laplacian-of-Gaussian (LoG) filtered image where sharp changes in the image are highlighted, as shown in Fig. S5 (**f**). To solve Equation (3), we equate the derivative of Equation (2) with respect to *A*, *c* to zero, which yields:

$$\hat{A}[\mathbf{k}] = \frac{\sum_{x \in S} f[\boldsymbol{k} - \boldsymbol{x}] g[\boldsymbol{x}] - (\sum_{x \in S} g[\boldsymbol{x}])(\sum_{x \in S} f[\boldsymbol{k} - \boldsymbol{x}])/p}{\sum_{x \in S} g[\boldsymbol{x}]^2 - (\sum_{x \in S} g[\boldsymbol{x}])^2/p} \tag{4}$$

$$\hat{c}[\mathbf{k}] = \frac{\sum_{x \in S} f[\boldsymbol{k} - \boldsymbol{x}] - \hat{A}[\mathbf{k}] \sum_{x \in S} g[\boldsymbol{x}]}{p} \tag{5}$$

where *p* is the total pixel number in *S*. A representative denoised image obtained through this process is shown in Fig. S5 (**d**). The identification on significant signals is achieved by performing a *t*-test on each of the candidate signals:

$$T(\boldsymbol{k}) = \sqrt{p}\frac{\hat{A}[\boldsymbol{k}] - \kappa\sigma_n[\boldsymbol{k}]}{\sqrt{\sigma_A^2[\boldsymbol{k}] + \kappa^2 s_n^2[\boldsymbol{k}]}} \tag{6}$$

where $\kappa = \sqrt{2}\,\mathrm{erf}^{-1}(1 - 2\alpha)$, α is the significance level ($\alpha < 0.01$ in the data analysis), σ_A is the uncertainty on $\hat{A}[\boldsymbol{k}]$ obtained from error propagation, and s_n is the uncertainty on σ_n from the standard error of variance. By selecting the pixels whose p-values are lower than α, we can obtain the mask of significance highlighting the centers of scattering signals, as shown in Fig. S5 (**e**). Finally, by overlaying the mask of significance with the local maximum of the Gaussian-of-Laplacian filtered image, the centroid of each scattering signal can be effectively captured, as indicated in Fig. S5 (**g**).

(3) Following the detection of scattering signals within each frame, a single-particle tracking algorithm (μ-track) was utilized to obtain the trajectory information based on the previously obtained centroid coordinates and frame number[4]. Here, we assume that one signal can at most link to one signal in another frame, and no merging or splitting is allowed. A typical image containing AuNP trajectories is shown in Fig. S5 (**h**).

Supplementary Note 2: Temporal Couple-Mode Theory

As shown in Fig. S8, the photonic crystal (PC) assisted nanoparticle (NP) scattering process can be modelled via a temporal couple-mode theory (TCMT)[5,6]. Considering a PC cavity (resonant frequency ω_0, with radiative decay rate γ_r) that exchanges energy with incoming ($s_{(1,2)+}$) and outgoing ($s_{(1,2)-}$) waves ($s_{2+} = 0$) with coupling constant $\kappa_{(1,2)}$, $d_{(1,2)}$ respectively. Additional NP scattering channel is characterized with scattering damping rate γ_{sc} (Supplementary Fig. 1). We first describe the behavior of the resonator

$$\frac{d}{dt}A = -i\omega A = (-i\omega_0 - \gamma_r - \gamma_{sc})A + \kappa s_{1+} \tag{7}$$

$$\begin{pmatrix} s_{1-} \\ s_{2-} \end{pmatrix} = \begin{pmatrix} 0 & 1 \\ 1 & 0 \end{pmatrix} \begin{pmatrix} s_{1+} \\ 0 \end{pmatrix} + A \begin{pmatrix} d_1 \\ d_2 \end{pmatrix} \tag{8}$$

where A is the amplitude of the PC cavity mode, and κ is the coupling efficiency between the PC cavity and the external waves. For simplicity, we assume mirror symmetry in the system (which is an approximation for our PC design) therefore $d_1^2 = d_2^2$. From energy conservation and time-reversal symmetry, we have $\kappa = d_1 = -d_2 = \sqrt{1/\gamma_r}$. From Equation (7) and Equation (8), the reflection and transmission coefficient are obtained as

$$R = \frac{|s_{1-}|^2}{|s_{1+}|^2} = \frac{\gamma_r^2}{(\omega - \omega_0)^2 + (\gamma_r + \gamma_{sc})^2} \tag{9}$$

$$T = \frac{|s_{2-}|^2}{|s_{1+}|^2} = \frac{(\omega - \omega_0)^2 + \gamma_{sc}^2}{(\omega - \omega_0)^2 + (\gamma_r + \gamma_{sc})^2} \tag{10}$$

and the scattered power is then obtained as

$$1 - R - T = \frac{P_{sc}}{P_{in}} = \frac{2\gamma_r \gamma_{sc}}{(\omega - \omega_0)^2 + (\gamma_r + \gamma_{sc})^2} \tag{11}$$

which describes the resonance-assisted scattering phenomenon. The total quality factor of the scattering influenced PCGR mode now becomes

$$Q_E = \frac{\omega_0}{2(\gamma_r + \gamma_{sc})} = (Q_r^{-1} + Q_{sc}^{-1})^{-1} \tag{12}$$

where

$$Q_r = \frac{\omega_0}{2\gamma_r}, Q_{sc} = \frac{\omega_0}{2\gamma_{sc}} \tag{13}$$

In general, for a single layer of NPs, the solitary scattered power can be modelled as

$$P_{sc}^{S} = (N_0 \sigma_{sc} d) \times P_{in} \tag{14}$$

where N_0 is the number density of NPs, σ_{sc} is the NP scattering cross section, P_{in} is the pump power, while d is the NP diameter. From Equation (11) and Equation (14) we can obtain the PC enhancement on the scattered power by

$$\Lambda(\omega_0) = \frac{P_{sc}}{P_{sc}^{S}} = \frac{2\gamma_r \gamma_{sc}}{(\gamma_r + \gamma_{sc})^2} \frac{1}{N_0 \sigma_{sc} d} = \frac{2\lambda_0 \alpha}{\pi n d_e} \frac{Q_E^2}{Q_r} \tag{15}$$

where P_{sc} and P_{in} are respectively the scattered power and the incident power, λ_0 is the resonant wavelength, α is the energy confinement of the PC mode in the NP layer (dependent on the size and material of the particle, ~12% for AuNPs of 20 nm in diameter), n is the refractive index of water and d_e is the effective length of the evanescent field (~ 100 nm). In general, as the NP grows larger in size, the corresponding perturbation in the pristine cavity mode becomes more significant. In other words, the total quality factor Q_E of the scattering influenced PCGR mode deteriorates with larger NPs, leading to the diminishing scattering enhancement factor as obtained from FEM simulation shown in Fig. 4b.

Supplementary Note 3: Numerical Simulations

The PC transmission/reflection spectrum and the band structure was calculated using both three-dimensional FEM and FDTD simulations. The geometric parameters of the PC cavity ($\Lambda = 390$ nm, $f_1 = 0.60$, $f_2 = 0.55$, $f_3 = 0.74$, $d_1 = 97$ nm, $d_2 = 67$ nm, $d_3 = 100$ nm) were measured from the SEM image of a fabricated sample, as shown in Fig. S1. The refractive index of TiO_2 was taken from Siefke[7] while the refractive indexes of the glass substrate ($n_{glass} = 1.510$) and the UV curable epoxy ($n_{epo} = 1.464$) were referenced from the manufacturers. The unit cell in simulation consisted of one primitive PC period in the transverse direction imposed with Floquet periodic boundary conditions (with additional perfectly matched layer (PML) boundaries

imposed in the vertical direction for FEM calculation), and the excitation field was launched from the top port (of the water layer) with transverse magnetic polarization at a incident angle defined as $\theta_i = \text{asin}(n_{air} \sin\theta \,/n_{water})$, where θ is the incident angle defined with respect to air.

The radiated power distribution of a dipole on the PC/glass substrate was obtained using 2D FEM simulation. The extended PC structure was approximated by 9 periods of PC, and the vertically orientated electric dipole was located at the center of the central PC ridge with 0.5 nm separation from the surface.

For FEM simulation of the cavity-enhanced scattering, the AuNP was modelled as a spherical structure with the refractive index provided by Johnson and Christy[8]. The mesh size at the NP was smaller than one tenth of the particle diameter, as shown in Fig. S6a. Further reduction on the mesh size to one twentieth of the particle diameter showed marginal improvement on the accuracy of near-field profile (Fig. S6). Using the wave optics module, the full field solution under Floquet periodic conditions was first calculated to simulate the field profile of a pristine extended PC cavity, which was then used as the background field for AuNP excitation and the previous Floquet periodic boundary conditions were replaced by PML boundary conditions to prevent the back-scattered field from the computational boundary. The scattering cross section can then be calculated as

$$\sigma_{\text{sc}} = \frac{1}{I_0}\iint_{S_{np}} (\boldsymbol{n}\cdot\boldsymbol{S_{sc}})dS \tag{16}$$

where I_0 is the incident intensity, S_{np} is the closed surface of the AuNP, $\boldsymbol{n}$ is the normal vector on the surface and $\boldsymbol{S_{sc}}$ is the relative Poynting vector. Similarly, the scattering cross section of

AuNP on the glass substrate is calculated under the same condition with the PC structure removed.

It is noteworthy that the PC enhancement on the AuNP scattering is dependent on the relative NP location within one PC period. As shown in Fig. S7, the edge of the PC ridge provides more scattering enhancement due to the higher near-field intensity of the PCGR mode at the edge. This location sensitivity explains the slightly broadened PRISM signal distribution for AuNPs in comparison with the DLS measurement results. The scattering enhancement at the center of the PC ridge is close to the averaged enhancement value, and is used for the discussion on the PC-assisted scattering process in the article.

Supplementary References

1 Aguet, F., Antonescu, C. N., Mettlen, M., Schmid, S. L. & Danuser, G. Advances in analysis of low signal-to-noise images link dynamin and AP2 to the functions of an endocytic checkpoint. *Developmental cell* **26**, 279-291 (2013).

2 Lee, I.-B. *et al.* Interferometric scattering microscopy with polarization-selective dual detection scheme: Capturing the orientational information of anisotropic nanometric objects. *ACS Photonics* **5**, 797-804 (2017).

3 Patterson, G., Davidson, M., Manley, S. & Lippincott-Schwartz, J. Superresolution imaging using single-molecule localization. *Annual review of physical chemistry* **61**, 345-367 (2010).

4 Jaqaman, K. *et al.* Robust single-particle tracking in live-cell time-lapse sequences. *Nature Methods* **5**, 695-702 (2008).

5 Regan, E. C. *et al.* Direct imaging of isofrequency contours in photonic structures. *Science Advances* **2**, e1601591 (2016).

6 Zhen, B. *et al.* Enabling enhanced emission and low-threshold lasing of organic molecules using special Fano resonances of macroscopic photonic crystals. *Proceedings of the National Academy of Sciences* **110**, 13711-13716 (2013).

7 Siefke, T. *et al.* Materials pushing the application limits of wire grid polarizers further into the deep ultraviolet spectral range. *Advanced Optical Materials* **4**, 1780-1786 (2016).

8 Johnson, P. B. & Christy, R.-W. Optical constants of the noble metals. *Physical review B* **6**, 4370 (1972).
