# Supplementary material for: Photonic resonator interferometric scattering microscopy
Source: Nat Commun. 2021 Mar 19;12:1744. doi: 10.1038/s41467-021-21999-3 (PMC7979857; doi:10.1038/s41467-021-21999-3)
Supplement: Supplementary file 2 — Description of Additional Supplementary Files [file 41467_2021_21999_MOESM2_ESM.pdf]

Description of Additional Supplementary Files

Supplementary Video 1. Exemplary video of raw images, interferometric scattering signals and NP detection results. This movie corresponds to the observation of AuNPs (40 nm in diameter), recorded at the 600 frames per second (PFS) over 10 s at 25 W/cm^2. Left panel: Streaming of the raw images captured by the camera. Middle panel: processed interferometric scattering images highlighting the Brownian motion driven AuNPs. Right panel: detected centroids of scattering signals. Scale bar: 2 µm.

Supplementary Video 2. PRISM detection on SARS-CoV-2 virions. Direct observation of SARS-CoV-2 virions suspended in PBS solution is recorded at the 600 frames per second (PFS) over 10 s at 25 W/cm^2. Scale bar: 2 µm.
